# Health system costs of rheumatic heart disease care in South Africa

**DOI:** 10.1186/s12889-021-11314-6

**Published:** 2021-07-03

**Authors:** Assegid G. Hellebo, Liesl J. Zuhlke, David A. Watkins, Olufunke Alaba

**Affiliations:** 1 Health Economics Unit, Faculty of Health Sciences, School of Public Health and Family Medicine, University of Cape Town, Cape Town, South Africa; 2 Division of Paediatric Cardiology, Department of Paediatrics, Faculty of Health Sciences, Red Cross War Memorial Children’s Hospital, University of Cape Town, Cape Town, South Africa; 3 Division of Cardiology, Department of Medicine, Faculty of Health Sciences, Groote Schuur Hospital, University of Cape Town, Cape Town, South Africa; 4 Division of General Internal Medicine, Department of Medicine, School of Medicine, University of Washington, Seattle, USA; 5 Department of Global Health, University of Washington, Seattle, USA

**Keywords:** Unit cost, Cost analysis, Provider cost, Rheumatic fever, Rheumatic heart disease, Tertiary care

## Abstract

**Background:**

Rheumatic Heart Disease (RHD) is a disease of poverty that is neglected in developing countries, including South Africa. Lack of adequate evidence regarding the cost of RHD care has hindered national and international actions to prevent RHD related deaths. The objective of this study was to estimate the cost of RHD-related health services in a tertiary hospital in the Western Cape, South Africa.

**Methods:**

The primary data on service utilisation were collected from a randomly selected sample of 100 patient medical records from the Global Rheumatic Heart Disease Registry (the REMEDY study) - a registry of individuals living with RHD. Patient-level clinical data, including, prices and quantities of medications and laboratory tests, were collected from the main tertiary hospital providing RHD care. All annual costs from a health system perspective were estimated in 2017 (base year) in South African Rand (ZAR) using a combination of ingredients and step-down costing approaches and later converted to United States dollars (USD). Step-down costing was used to estimate provider time costs and all other facility costs such as overheads. A 3% discount rate was also employed in order to allow depreciation and opportunity cost. We aggregated data to estimate the total annual costs and the average annual per-patient cost of RHD and conducted a one-way sensitivity analysis.

**Results:**

The estimated total cost of RHD care at the tertiary hospital was USD 2 million (in 2017 USD) for the year 2017, with surgery costs accounting for 65%. Per-patient, average annual costs were USD 3900. For the subset of costs estimated using the ingredients approach, outpatient medications, and consumables related to cardiac catheterisation and heart valve surgery were the main cost drivers.

**Conclusions:**

RHD-related healthcare consumes significant tertiary hospital resources in South Africa, with annual per-patient costs higher than many other non-communicable and infectious diseases. This analysis supports the scaling up of primary and secondary prevention programmes at primary health centers in order to reduce future tertiary care costs. The study could also inform resource allocation efforts and provide cost estimates for future studies of intervention cost-effectiveness.

**Supplementary Information:**

The online version contains supplementary material available at 10.1186/s12889-021-11314-6.

## Background

In South Africa, private and public health systems exist in parallel. The public health system services over 80% of the population [1]. Currently, the public health system is largely funded through allocations from general tax revenue and small contributions acquired from the local government revenue and user charges [1, 2]. However, the country’s public health sector continues to face many challenges - ranging from a shortage of resources to a growing and ageing population and the high burden of diseases [1–3]. Mainly, non-communicable diseases (NCDs) have become a public health issue requiring urgent attention. One preventable and treatable but neglected form of NCD is rheumatic heart disease (RHD) [4].

Rheumatic heart disease (RHD) is the most common acquired cardiovascular disease among children and adolescents in low- and middle-income countries [5]. It is a chronic inflammatory disease of the heart valves that results from recurrent episodes of acute rheumatic fever (ARF). RHD often leads to heart failure, stroke, and premature death, including among children and young adults [5, 6]. A recent study estimated about 33 million prevalent cases and 320,000 deaths from RHD globally in 2015 [6]. African countries have some of the highest rates of RHD in the world [6, 7].

Previous studies from higher-income regions suggest that RHD is a high-cost disease because of its clinical complexity fuelled by frequent and lengthy hospital admissions and expensive surgical care; as well as the significant physical and psychological impact on patients and households [8]. The condition results in high levels of resource use, particularly in middle-income countries where access to advanced cardiovascular services, including heart valve surgery, is increasing [8, 9]. Even though surgery can save or prolong life, access remains low in many countries due to shortages in surgical services themselves as well as a lack of early diagnosis and prompt referral [10]. It is also unclear whether cardiac surgery programmes are fiscally sustainable in low- and middle-income countries [5, 11].

Lack of information on the cost of RHD is a barrier to assessing and distributing an equitable allocation of resources between RHD and other diseases and to analysing the cost-effectiveness of new RHD intervention or prevention strategies compared to existing strategies [12]. A recent review found very few studies on the economics of RHD and no studies of the cost of care in the African context [9–12]. The objective of the present study was to estimate the cost of care for RHD from the public health system (provider) perspective at a tertiary hospital in South Africa. We look at outpatient costs and inpatient costs at four different levels of care – outpatient care, cardiac intensive unit (ICU) care, cardiac catheterisation laboratory, and cardiac surgical theatre care – that are the major clinical areas/levels where individuals with RHD receive cardiac services (and incur costs) in the South African context.

## Methods

### Study setting and population

This cross-sectional cost analysis study was carried out in 2017 at Groote Schuur Hospital (GSH), a public tertiary level healthcare facility that serves the Western Cape Province. We focused on estimating (a) the total costs incurred at GSH in 2017 because of RHD and (b) the per-patient annual cost of care using a representative sample of individuals requiring tertiary care at GSH. The RHD Pathway of Care (presented in Additional file 1: Appendix 1) is often different, and most cases are referrals from outside GSH.

### Identifying clients with RHD

In this study, cost data from GSH were complemented with more detailed data on patient utilisation of health services. These were obtained from randomly selected participants (*n* = 100) in the Global Rheumatic Heart Disease Registry (the REMEDY study), an international registry of RHD that includes cases enrolled at GSH [13].

The REMEDY study recruited 448 participants from GSH over 2012–2014. Research staff reviewed and selected new patient folders, separately documenting current patterns of disease pathology and care delivery in order to investigate the clinical epidemiology of RHD. The observational REMEDY study did not provide any care to RHD patients; however, they captured major adverse events and instances of defaulting on medications or other aspects of care over a 24-month follow-up. We randomly sampled 100 of the 448 REMEDY participants in order to calculate parameters related to healthcare utilisation.

It is important to note that subjects who enrolled in REMEDY rarely utilised emergency departments (ED) because they were generally clinically stable, visiting GSH outpatient departments twice yearly on average to evaluate progression. In addition, pharmacy (refill) visits are few and are much less resource-demanding compared to clinical follow-ups; often, patients collect pre-packed and dispensed medications from a third-party provider. This situation made it challenging to defining the input and outcome guides for ED and pharmacy encounters at the individual, population and system levels. Consequently, we did not attempt to estimate pharmacy visit or emergency department costs outside GSH.

### Costing approach

We calculated the economic cost of providing RHD care from the health system (provider) perspective. Retrospective costing was conducted using a mix of the ingredients and step-down approach by aggregating costs at different levels. All data were collected from January 2017 to December 2017. Costs were calculated in ZAR using 2017 as the base year and later converted to USD. We used a set of four data collection instruments, one for each of the major clinical units where RHD care is delivered at GSH: cardiac clinic, medical and ICU ward, cardiac catheterisation laboratory, and surgical theatre.

### Ingredients costing

This approach primarily used detailed medical records and observations to measure the specific resources required for delivering RHD care. We studied utilisation patterns of resources such as medications, laboratory tests, and diagnostics and consumables related to RHD care. For example, the cost of medication was estimated according to the number of medications recorded as dispensed for each of the 100 REMEDY participants then multiplied by the cost per dosage/injection. We also reviewed REMEDY and other hospital medical records to establish utilisation rates for specific services, and semi-structured interviews with clinical experts in RHD care were also conducted. The cost-share of cardiac medical and ICU care on RHD was calculated using the number of inpatient bed-days at GSH; i.e. by multiplying the number of admissions with the average length of stay. The average length of stay of individuals with RHD in the cardiac wards was 4.7 days per admission according to the most recent annual report.

We obtained pricelists for commodities from the Western Cape Department of Health (Wendy Braynt, 2018 - personal communication, 8 November). As per treatment guidelines and the data obtained from medical records, direct medical costs were calculated using the accounting identity; i.e. costs incurred are the product of prices and quantities of goods and services consumed.

### Step-down costing

Step-down costing approach is commonly used to estimate costs that are long-term but not directly related to the patient utilisation, including costs borne by support departments in health facilities [14]. Capital costs (e.g. medical building, equipment and furniture) and recurrent indirect costs (e.g. overhead costs incurred by GSH, electricity, water, cleaning, security) of proving RHD care were costed using the step-down approach.

Capital item costs and useful lifetimes were obtained from the hospital procurement department. Original costs obtained were also inflated to 2017 levels using the consumer price index [14]. Equipment and furniture item costs were apportioned to RHD care according to the share of RHD patients using the items. Building costs were estimated using a square meter of space apportioned for RHD care. Costs were calculated by multiplying by the building replacement value per square meter (USD 3200), which was obtained from the building and engineering company-approved tender estimate for GSH [15]. Recurrent cost data were obtained from the finance department of GSH for the 2016/2017 financial year.

As recommended by the World Health Organization (WHO), capital items were discounted and annualised using a discount factor of 3%, reflecting depreciation and the opportunity cost of purchasing the capital items [16]. An estimated lifetime of 30 years for buildings and ten years for equipment and furniture were used. Sensitivity analyses were conducted using 0, 5 and 10% (which is a government bond rate in South Africa) discount rates to verify the robustness of the results [16, 17].

Costs were categorised into overheads (i.e. not directly associated with caring) and final service centers (i.e. used in care) [18]. Overhead costs comprise the cost of general support services departments that are important for the facility to operate [14, 16]. These include utilities (i.e. electricity, water), non-clinical personnel (i.e. cleaning, security) as well as stores that provide non-patient specific services [16, 17]. Overhead costs were estimated by assessing the overhead expenditures, assuming that all patients utilised equal amounts of overhead costs per unit time when they visit GSH [17, 18].

The calculation for overhead costs per visit was the annual overhead expenditure divided by the annual number of patient visits. This approach was used for both outpatient and inpatient departments of GSH because the cost centre accounting system does not exist in the hospital. Overhead expenditures were allocated directly to outpatients and inpatient departments using an allocation factor based on the patient day equivalent (PDE) concept [17]. PDE is estimated as:

$$ PDEoutpatients=\left( annual\ inpatient\ days\right)\times \frac{1}{weighting\ factor}\Big)+\left( annual\ outpatient\ visits\right) $$

$$ PDEinpatients=\left( annual\ inpatient\ days\right)+\left( annual\ outpatient\ visits\times weighting\ factor\right) $$

The local practice at the University of Cape Town is to employ the (empirically-based) rule of thumb that an outpatient visits costs one-third (1/3 = 0,33) of an inpatient day, and this is used as a weighing factor [17]. Overhead costs per outpatient visit and inpatient day at GSH are therefore expressed as:

$$ \mathrm{Overhead}\ \mathrm{cost}\ \mathrm{per}\ \mathrm{outpatient}\ \mathrm{visit}=\frac{\mathrm{annual}\ \mathrm{overhead}\ \mathrm{expenditure}}{{\mathrm{PDE}}_{outpatients.}} $$

$$ \mathrm{Overhead}\ \mathrm{cost}\ \mathrm{per}\ \mathrm{inpatient}\ \mathrm{day}=\frac{annual\ overhead\ expenditure}{PDE_{inpatients}} $$

Costs of different centers within GSH that render services to the entire hospital (i.e. intermediate costs) were incorporated in overhead costing; these included the pharmacy and laboratory departments [18]. These intermediate and overhead costs were later added to the final cost department in a sequential manner. Recurrent and capital costs were also allocated to the four clinical units directly according to their actual resource utilisation. For instance, estimates of square meters of space used directly for care were used to distribute the building costs to the four units where RHD care is provided. Incurred recurrent and capital costs were distributed according to the clinical unit using allocation bases and resource usage. The number of individuals with RHD as a percentage of the total number of patients served in each clinical unit was used to calculate a utilisation factor reflecting the share of costs allocable to RHD.

There was no financial cost incurred by GSH for training nurses or other staff personnel specific to RHD; however, lectures were given by senior registrars to nursing staff every Tuesday; nurses also had an opportunity to participate in catheterisation procedures to teach them about post-procedure care. We excluded the economic cost component of these lectures.

### Personnel costing

Statistics regarding time allocation and time spent by different personnel providing care or consulting patients with specific diagnoses such as RHD are not available at GSH. We obtained information from key clinical/hospital informants and ward operational managers through unstructured interviews and review of staff weekly duty timesheets. Eight non-participatory observations were conducted to understand patient management and staff time allocation and to identify and examine costs involved.

Total annual salary grades were obtained from the most recent GSH financial records and divided to the number of productive minutes. The RHD specific personnel costs were calculated depending on the average amount of time each staff member spent on patients which were established by non-participatory observations. The limitation of this strategy is that responses could not be authenticated through time-motion studies; hence it might over- or underestimate the share of time spent on providing RHD related care.

### Data analysis

Study data were entered into Microsoft Excel 2016 for quality evaluation. This Excel costing tool is provided as a supplementary appendix file. STATA 14 was used for the analysis of REMEDY data (utilisation and patient demographic characteristics) (Statacorp, 2015).

Total cost was calculated by adding the ingredients and step-down cost estimates, while the total cost per admission was estimated by multiplying the average cost per inpatient day by the average length of stay. The annual cost of care per-patient at each of the four clinical units was also calculated as the product of the unit cost (per encounter) times the annual utilisation rate. The overall annual cost of care per patient was calculated as the sum of annual costs at each of the four clinical units. Where the hospital provided Outpatient Clinic care for 668 patients (with a total of 7423 visits), Medical and ICU care for 309 patients (with 1227 hospitalisation days), Catheterisation Laboratory cares for 301 patients (with 1426 Cath lab admissions), and Surgical Theatre for 56 patients (with 294 procedures). For comparison, the total cost of providing RHD care at GSH in 2017 was extracted from the spreadsheets as the aggregate costs at each of the four clinical units (i.e. the costs required to deliver care for all individuals with RHD, including but not limited to the 100 REMEDY participants).

In order to assess the impact that changes in assumptions or parameters would have on final cost estimates; one-way deterministic sensitivity analyses were performed. The following cost model inputs were varied: (a) discount rate of 0, 5 and 10% on capital costs [19]; and (b) utilisation rates of the four clinical units were varied from base values to lower and upper 95% confidence interval estimates (per the REMEDY database) to examine their effect on average annual per-patient costs [17, 19].

### Summary of cost categorisation

Costs were categorised as recurrent and capital (fixed). Variable costs were also expressed as recurrent costs. Fixed costs comprised costs of buildings, equipment and furniture, while recurrent costs were personnel salaries, materials and consumables, overheads (i.e. electricity, water, telephone, sewage, laundry, security), laboratory tests, blood transfusions and medications.

## Results

### Baseline characteristics of the study sample

The demographical characteristics of the REMEDY study participants are provided in Table 1. The majority of the participants were female (67%), with the current age of participants ranging 24–81 years. Participants were predominately from low-income backgrounds, and most were unemployed. Forty-eight percent had valve surgery at some point in their lifetime, ranging between 1980 and 2016.

**Table 1:** Demographical characteristics of patients (*n* = 100) with RHD whose medical records were used for this study

Characteristic	Estimate
Mean age	48 years
Female sex	67%
Employment history	Employed 17% Self-employed 1% Unemployed 83%
Educational attainment	No formal education 2% Primary level 3% Secondary level 81% Tertiary level 4%

The REMEDY database provided the number of visits and admissions for the 100 patients that attended cardiac care at GSH. Utilisation data were collected over two consecutive years to capture longer-term rates in clinically stable patients who only attend outpatient clinics (i.e., less frequently); these parameters are provided in Table 2.

**Table 2:** Average annual utilisation rates for RHD care at four clinical units at GSH

Clinical unit	Outpatient clinic	Medical and intensive care unit (ICU)	Cath lab	Surgery/theatre
**Per-patient utilisation rate per year (95% confidence interval)**	2.9 (2.0–3.9)	0.31 (0.11–0.50)	0.19 (0.068–0.31)	0.22 (0.043–0.39)

### The unit cost of RHD services by clinical unit

Summaries of average annual per-patient costs for the four different clinical units (outpatient, cardiac ICU and ward, cardiac catheterisation laboratory, and surgical theatre) are provided in Table 3, Table 4, Table 5 and Table 6, respectively. On a per-unit basis, the cardiac medical and ICU costs comprised a smaller proportion of RHD costs than did the cardiac catheterisation laboratory and surgical theatre costs. Among cost components estimated using the ingredients approach, medications and staff costs were the drivers of total costs of outpatient visits, while blood transfusions and staff costs in inpatient medical and ICU care were the highest. Consumables costs were the drivers in the cardiac catheterisation lab and surgical theatre.

**Table 3:** Outpatient total and unit costs

Cost component	Annual total cost to GSH in 2017 (US$)	Unit cost per encounter in 2017 (US$)
**Costs obtained through step-down approach**		
**Personnel**	$ 170,000	$ 55
**Overheads**	$ 20,000	$ 5.4
**Maintenance**	$ 2300	$ 1.7
**Consumables**	$ 8000	$ 2.3
**Building**	$ 25,000	$ 6.6
**Equipment & furniture**	$ 11,000	$ 2.9
***Step-down sub-total***	$ 240,000	$ 76
**Costs obtained through ingredients approach**		
**Medications**	$ 160,000	$ 240
**Laboratory tests**	$ 23,700	$ 35
***Ingredients sub-total***	$ 170,000	$ 250
**Total cost**		
***Sum of sub-totals***	$ 410,000	$ 320

Note: totals may differ slightly from individual rows due to rounding

**Table 4:** Cost of cardiac medical and intensive care unit care

Cost component	Annual total cost to GSH in 2017 (US$)	Unit cost per encounter in 2017 (US$)
**Costs obtained through step-down approach**		
**Personnel**	$ 93,000	$ 50
**Overheads**	$ 31,300	$ 17
**Maintenance**	$ 3700	$ 2.0
**Consumables**	$ 23,400	$ 13
**Building**	$ 36,000	$ 20
**Equipment & furniture**	$ 3000	$ 7.2
***Step-down sub-total***	$ 190,000	$ 110
**Costs obtained through ingredients approach**		
**Medications**	$ 9700	$ 32
**Blood transfusions**	$ 14,000	$ 45
**Laboratory tests**	$ 26,000	$ 80
***Ingredients sub-total***	$ 49,000	$ 160
**Total cost**		
***Sum of sub-totals***	$ 240,000	$ 270

Note: totals may differ slightly from individual rows due to rounding

**Table 5:** Cost of catheterisation laboratory care

Cost component	Annual total cost to GSH in 2017 (US$)	Unit cost per encounter in 2017 (US$)
**Costs obtained through step-down approach**		
**Personnel**	$ 91,000	$ 290
**Maintenance**	$ 3100	$ 10
**Consumables**	$ 310,000	$ 970
**Building**	$ 30,000	$ 100
**Equipment & furniture**	$ 22,000	$ 430
***Step-down sub-total***	$ 450,300	$ 1800
**Costs obtained through ingredients approach**		
**Medications**	$ 39,000	$ 130
***Ingredients sub-total***	$ 40,000	$ 130
**Total cost**		
***Sum of sub-totals***	$ 490,000	$ 1900

Note: totals may differ slightly from individual rows due to rounding

**Table 6:** Cost of cardiac surgical theatre care

Cost component	Annual total cost to GSH in 2017 (US$)	Unit cost per encounter in 2017 (US$)
**Costs obtained through step-down approach**		
**Personnel**	$ 210,000	$ 2300
**Maintenance**	$ 4600	$ 50
**Consumables: general**	$ 62,000	$ 680
**Consumables: perfusion**	$ 250,000	$ 2800
**Consumables: anaesthesia**	$ 19,000	$ 200
**Consumables: prosthetic valves**	$ 91,000	$ 1000
**Building**	$ 46,000	$ 500
**Equipment & furniture**	$ 79,000	$ 3100
***Step-down sub-total***	$ 750,100	$ 11,000
**Costs obtained through ingredients approach**		
**Medications**	$ 45,000	$ 800
***Ingredients sub-total***	$ 45,000	$ 800
**Total cost**		
***Sum of sub-totals***	$ 800,000	$ 12,000

Note: totals may differ slightly from individual rows due to rounding

The cost of routine medications was the major cost driver in outpatient clinic care (74%) followed by staff time (17% of the cost). In the catheterisation lab and surgical theater, consumables (such as prosthetic valves and specialized catheters) were the major drivers (51 and 44%, respectively).

In 2016 the cardiac surgery theatre at GSH performed 322 valve replacements, 56 of which were RHD-related. The most common valve replacement types were aortic (46%), mitral (41%), and mixed aortic and mitral (13%).

### Aggregate cost of RHD care

As seen in Table 7, RHD care cost GSH an estimated US$ 2 million in 2017. The average cost per patient-year, based on utilisation data from the REMEDY study, was an estimated US$ 3900 with (65%) of the total cost being driven by surgical theatre cost. In GSH, Forty-eight percent of the participant sample had valve surgery at some point in their lifetime, ranging 1980 to 2016. Consumables related to cardiac catheterisation and heart valve surgery were the main cost drivers. Outpatient care cost also accounts for a quarter of the total annual RHD care burden with medications (USD239) and personnel time (USD55) being the highest respectively.

**Table 7:** Summary costs: Aggregate costs to GSH in 2017 and estimated annual costs per-patient

Clinical unit	Aggregate cost to GSH in 2017 (US$)	% share of aggregate total cost	Estimated per-patient annual cost (US$)	% share of per-patient annual cost
**Outpatient clinic**	$ 400,000	21%	$ 930	25%
**Cardiac medical intensive care unit and ward**	$ 240,000	13%	$ 82	2.1%
**Cardiac catheterisation lab**	$ 490,000	26%	$ 360	9.4%
**Surgical theatre**	$ 800,000	41%	$ 2500	65%
**Total**	**$ 1,900,000**		**$ 3900**	

Note: percentages and sums may differ from totals due to rounding

### Sensitivity analyses

**Fig. 1:**
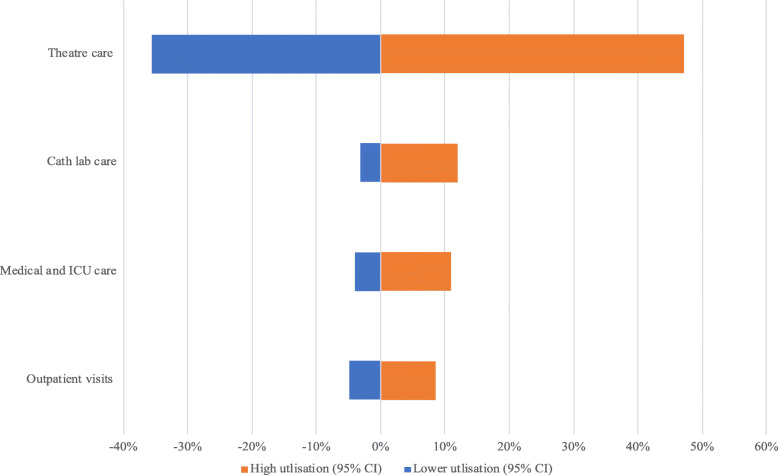
Effect of variation in utilisation rates on annual per-patient cost of RHD care

**Fig. 2:**
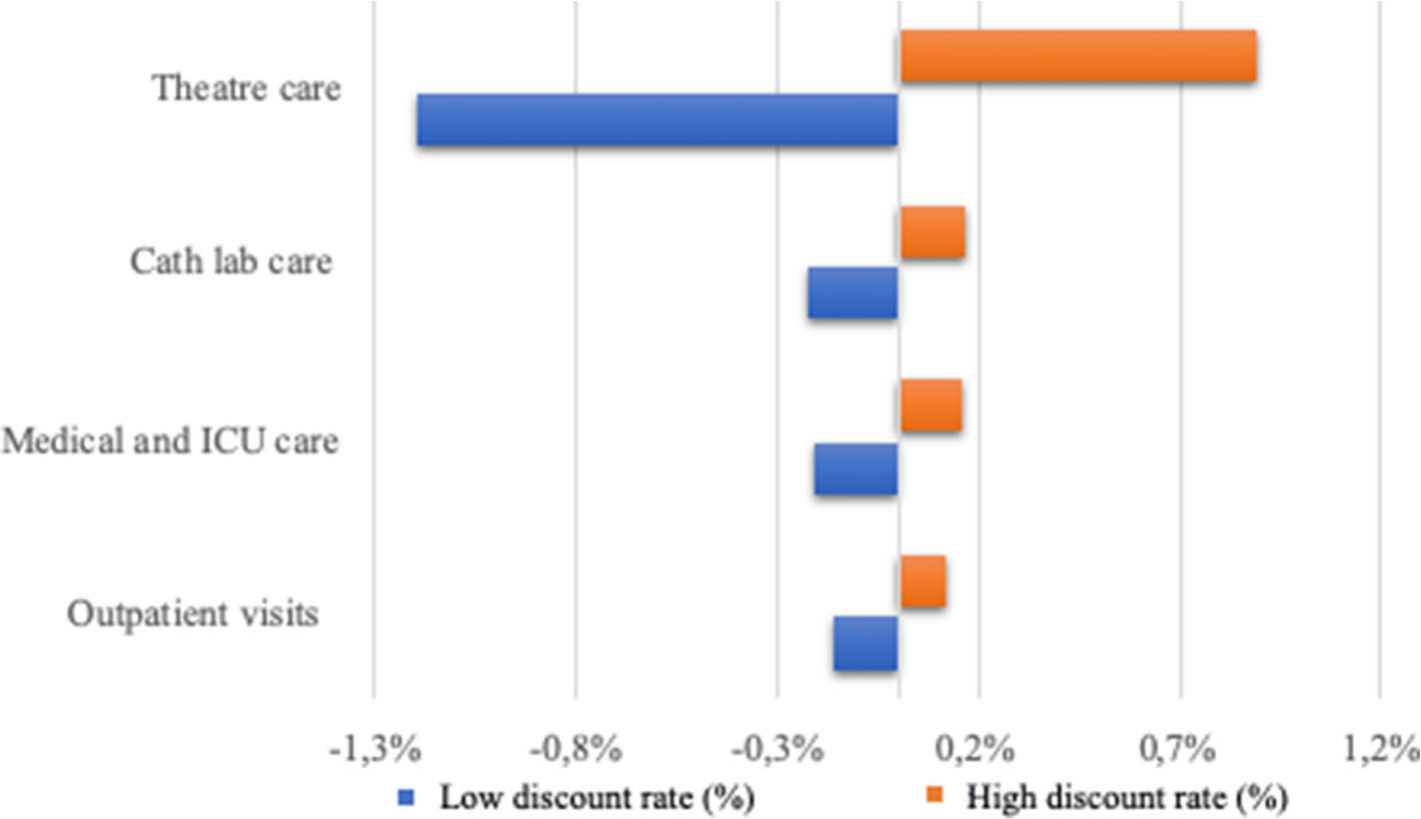
Effect of discount rate on annual cost of RHD care per patient

Figure 1 demonstrates the effects that varying utilisation rates at each of the four clinical units had on overall annual average per-patient costs. The costs were relatively less sensitive to variations in the discount rate (Fig. 2). In both sets of analyses, the influence of the parameters was largest on surgical theatre costs, since this was the most expensive clinical unit.

## Discussion

This cost analysis sought to estimate, from the provider perspective, the annual cost of RHD care in a tertiary centre in the Western Cape Province of South Africa. RHD was estimated to cost GSH about USD 1.9 million in the year 2017. These costs represent 11.2% of the total budget for GSH in the year 2017. Triangulating these estimates with data from the REMEDY registry, RHD was estimated to cost USD 3900 per patient per year in total, with most of the total cost being driven by surgical theatre and cardiac catheterisation laboratory costs. Previous studies indicated that the per-patient cost of RHD care in the United States of America in 2002 was approximately USD 6000 [20], while the per-patient cost in China in 2012 was approximately USD 4700 [21]. A study from Havana, Cuba also indicated that the cost of severe RHD care in the 1990s was approximately USD 6300 [22], while a hospital-based study from Pakistan’s largest tertiary care hospitals found that the average cost of care per patient was USD 1179 [23]. The cost per-patient in South Africa for merely inpatient care in 2010 was USD 2900 [24]. These costs are much higher than the annual cost of the facility-level care delivery of HIV/AIDS, drug sensitive tuberculosis, and hypertension, which have been estimated at around USD 660 (in 2016) [25], USD 250 (in 2015) [26], and USD 260 (in 2016) [27] per year, respectively. Not only has managing RHD cost more on a per-patient basis than all three of these conditions combined, but it has also been more neglected in national health policy discussions [28].

To the best of our knowledge, there have been no previous costing studies on RHD in Africa and, only one prior costing study in a low- and middle-income country setting. A study in Brazil looking at RHD, quantified per-patient direct costs of RHD and rheumatic fever from the provider perspective using the ingredients approach [29]. Even after adjusting to a common currency and year, the cost estimates in this study are higher than in the Brazilian study, probably due to advances in care (i.e. new technologies) and to the fact that health sector prices tend to rise faster than inflation. In addition, the present study took a more detailed look at surgical care, the most expensive aspect of RHD care.

This study assessed the cost of RHD to the public sector. It did not include ambulatory costs or admission costs from the private sector. It also did not look at patient costs, including so-called “direct non-medical costs” such as transport and food, which – from a societal perspective – are a significant share of healthcare costs in South Africa [19]. However, the direct medical costs were still substantial, and from a societal perspective, the cost of surgical care from the provider perspective is probably the most significant single driver of costs.

It should be noted that, amongst the “direct” costs estimated using the ingredients approach, the main cost contributors to outpatient RHD care were monthly medications followed by personnel cost, while the cost of laboratory tests, personnel and blood transfusions were major drivers for inpatient medical and ICU ward costs. These findings are similar to a study from Australia in which two-thirds of the cost of ICU care in tertiary hospitals was related to personnel [30]. Further studies predicted that consumables expenditure (in general) is likely to increase in the future in light of new innovations and therapies [31]. Along these lines, the cardiac catheterisation lab and surgical theatre consumables were found to be major drivers of cost in this study. One implication of this study is that research on developing cheaper, locally made prosthetic valves and catheters should be supported, and measures should be taken to support the production and export of such products to other African countries [32].

This study can also be viewed as an analysis of the economic consequences of inadequate prevention of rheumatic fever and RHD. RHD can be entirely prevented by addressing bacterial sore throat in children using primary health centre-based approaches. When this prevention window is missed, rheumatic fever develops and can lead to heart valve damage, which can require one or more surgeries over the lifetime of affected individuals. Several programmes in Latin America and the Caribbean demonstrated that healthcare costs from RHD declined by more than 90% when comprehensive prevention efforts were undertaken [23]. However, barriers such as poor access to primary care, shortage of skilled staff and poor public awareness about diagnosis and treatment of sore throats hindered wide adoption of primary prevention of the disease. The present study provides crucial evidence for the Western Cape Department of Health, and the National Department of Health to scale up RHD prevention efforts in South Africa.

Our study had a number of important limitations. Firstly, it was a hospital-based cost analysis, with the usual limitations of such a study design. One hundred patient records were reviewed; however, some details were missing, while those with severe disease were often presented with little detail. Nevertheless, considerable effort was taken to produce accurate and relevant estimates. Secondly, medication records did not disaggregate drugs related to RHD from drugs with other indications, potentially leading to over-estimates of RHD-specific medication costs. Thirdly, out of the 100-participant sample, only 88 individuals were consistently engaged in RHD care in GSH. This finding raises the question of whether they were receiving care elsewhere. As noted previously, for practical reasons, we were unable to gather pharmacy dispensing or emergency department costs. In total, these factors might lead to an under-estimate of per-patient costs and GSH costs. Finally, the step-down approach is not ideal for estimating some costs such as diagnostics (echocardiography, etc.) and pharmacy costs, better data would allow for more precise estimates of these costs using an ingredients approach. Notably, the cost of pharmacy services is likely to be higher at GSH than at health facilities in the community where most individuals with RHD receive the majority of their monthly prescriptions. Overall, our estimates of average annual per-patient care costs are probably higher compared to what they would have been if referring hospitals and clinics had been sampled.

## Conclusion

In summary, we found that RHD results in considerable costs to tertiary facilities in South Africa, a middle-income country with a high burden of disease and relatively adequate access to advanced cardiovascular care. Per-patient costs are much higher than other essential health conditions such as HIV/AIDS, tuberculosis, and hypertension. The high cost of treating RHD underscores the urgency of scaling up rheumatic fever prevention efforts in order to eliminate new cases of RHD eventually. Our study can serve as an input to future economic evaluations focused on RHD programmes and interventions in South Africa. We developed a costing framework and data collection tool (presented in Additional file 2: Appendix 2) that can be expanded to other aspects of RHD prevention and care as well as replicated in other African settings. The systematic and widespread collection of data on the cost of RHD will be a crucial component of raising awareness on the importance of the condition in Africa and of making a case for investing in RHD prevention.

## Supplementary Information

**Additional file 1.** Appendix 1: RHD Pathway of Care.

**Additional file 2.** The RHD costing spreadsheet.

## Data Availability

The datasets used and/or analysed during the current study are available from the corresponding author on reasonable request.

## Abbreviations

ARF: Acute Rheumatic Fever
GSH: Groote Schuur Hospital
ICU: Intensive Care Unit
RHD: Rheumatic Heart Disease
STATA: Statistics and Data Software Package
USD: United States Dollar
WHO: World Health Organisation
ZAR: South African Rand
