# Supplementary material for: Health system costs of rheumatic heart disease care in South Africa
Source: BMC Public Health. 2021 Jul 3;21:1303. doi: 10.1186/s12889-021-11314-6 (PMC8254987; doi:10.1186/s12889-021-11314-6)
Supplement: Supplementary file 1 — Additional file 1. Appendix 1: RHD Pathway of Care. [file 12889_2021_11314_MOESM1_ESM.docx]

# **Appendix 1:**

# **RHD Pathway of Care**

The cardiac clinic (E17) in GSH is the oldest devoted outpatient clinical cardiology service in sub-Saharan Africa. It provides care for cardiac patients (including RHD, ischemic heart disease, heart failure, endocarditis, and more) on average for about 7400 patients annually within GSH. Patients who are diagnosed with RHD come to E17 through different referral pathways such as emergency departments, primary care clinics, and other referral clinics within or outside GSH. Care in E17 is provided by senior registrars, consultants, and registered nurses, among others. RHD diagnoses are confirmed through detailed echocardiography as well as electrocardiograms and blood tests. Stable individuals with RHD are seen on one of three weekdays during which dedicated clinic sessions are held.

Patients with appointments for normal RHD follow-up care first collect their medical record folder from the receptionist then are seen by a nurse who performs routine screening activities and reviews the medical record. Depending on the appointment category (as indicated in the medical record evaluation) and the results from clinical measurements, patients are attended by a registered nurse, registrar, or consultant, or if there are severe or acute issues are referred to the emergency unit or admitted as inpatients at GSH (see below). Patients with RHD visit GSH as infrequently as twice a year if they are clinically stable. Routine appointments are scheduled along with one-month supply of medications and instructions to return in about 6-12 months unless complications arise. Follow-up appointments are planned to evaluate progression of disease and assess adherence to treatment. Annually, about 100 new patients with RHD are enrolled in E17.

Individuals who have complications of RHD may be admitted to GSH directly (in the case of emergencies) or on an elective basis (e.g., for expedited workup or a planned procedure). Additionally, some individuals may come directly to the emergency department (i.e., from the community setting) with acute symptoms that warrant stabilization in the hospital. While many RHD patients are admitted to the general wards some with complications are cared in the cardiac ward (C26) at GSH, a combined specialty acute care and intensive care ward. Some of these individuals may undergo cardiac catheterization (C25) or cardiac surgery (D21); these services are provided in units separate from C26.
